# Lethal Giant Larvae 1 Tumour Suppressor Activity Is Not Conserved in Models of Mammalian T and B Cell Leukaemia

**DOI:** 10.1371/journal.pone.0087376

**Published:** 2014-01-27

**Authors:** Edwin D. Hawkins, Jane Oliaro, Kelly M. Ramsbottom, Stephen B. Ting, Faruk Sacirbegovic, Michael Harvey, Tanja Kinwell, Jacques Ghysdael, Ricky W. Johnstone, Patrick O. Humbert, Sarah M. Russell

**Affiliations:** 1 Cancer Immunology Program, Peter MacCallum Cancer Centre, Melbourne, Victoria, Australia; 2 Sir Peter MacCallum Department of Oncology, The University of Melbourne, Melbourne, Victoria, Australia; 3 Stem Cell Research Group, Australian Centre for Blood Diseases, Monash University and Alfred Health, Melbourne, Victoria, Australia; 4 Cell Cycle and Cancer Genetics Laboratory, Peter MacCallum Cancer Centre, Melbourne, Victoria, Australia; 5 Institut Curie, Centre Universitaire, Bat 110 91405, Orsay, France; 6 Centre National de la Recherche Scientifique UMR 3306, Orsay, France; 7 INSERM (Institut National de la Santé et de la Recherche Médicale) U1005, Orsay, France; 8 Centre for Micro-Photonics, Swinburne University of Technology, Hawthorn, Victoria, Australia; University of Maastricht (UM), Netherlands

## Abstract

In epithelial and stem cells, lethal giant larvae (Lgl) is a potent tumour suppressor, a regulator of Notch signalling, and a mediator of cell fate via asymmetric cell division. Recent evidence suggests that the function of Lgl is conserved in mammalian haematopoietic stem cells and implies a contribution to haematological malignancies. To date, direct measurement of the effect of Lgl expression on malignancies of the haematopoietic lineage has not been tested. In Lgl1^−/−^ mice, we analysed the development of haematopoietic malignancies either alone, or in the presence of common oncogenic lesions. We show that in the absence of Lgl1, production of mature white blood cell lineages and long-term survival of mice are not affected. Additionally, loss of Lgl1 does not alter leukaemia driven by constitutive Notch, c-Myc or Jak2 signalling. These results suggest that the role of Lgl1 in the haematopoietic lineage might be restricted to specific co-operating mutations and a limited number of cellular contexts.

## Introduction

In *Drosophila*, the Scribble complex protein, Lethal giant larvae (Lgl) is a potent tumour suppressor. Lgl maintains epithelial cell polarity [1]–[3] and regulates asymmetric cell division (ACD), which produces unique daughter cells through the uneven distribution of cell fate determinant proteins during cell division. Homozygous Lgl mutant *Drosophila* develop epithelial tumours [4] and cooperate with Notch to drive epithelial neoplasia [5]. Importantly, Lgl function is conserved in mammals. Mice deficient in Lgl1 die neonatally from neuro-ectodermal tumours correlating with mislocalisation of the Notch regulator Numb, and consequent impaired ACD in neuronal precursors [6]. Lgl1^−/−^ mice also display neural tube hyperplasia associated with ectopic Notch signalling [6]. Providing further support for a possible role for Lgl in mammalian tumourigenesis, alterations in Lgl1 expression and copy number have been detected in a range of solid cancers in humans including glioblastoma, colorectal carcinoma, melanoma, prostate, breast and lung cancer [7]–[10]. Within the mammalian hematopoietic system, Notch signalling has been implicated in both the generation of haematopoietic stem cells (HSC), and in the regulation of their self-renewal [11], [12]. Additionally, the asymmetric localisation of Notch/Numb has been implicated in both normal HSC and leukemic daughter cell fate [13]. Notably, 50% of T-cell acute lymphoblastic leukaemia (T-ALL) patients possess mutations that constitutively activate Notch signalling [14] identifying regulators of this pathway as key components in leukaemia pathology.

Lgl1 has recently been described to affect cell cycle in mouse HSC, and gene signatures associated with Lgl1 deficiency in mouse HSC identify a group of cytogenetically normal AML with poor prognosis [15]. Additionally, a mutation in Lgl2 has been observed in one AML patient in the presence of additional co-operating mutations[16], suggesting that Lgl2 is not sufficient for leukemogenesis but might be a contributing factor. These data suggest that the tumour suppressor function of Lgl could be conserved in hematopoietic cells, and could be relevant to leukaemia. We therefore tested the function of Lgl1 by gene deletion in models of haematopoietic malignancies.

## Materials and Methods

### Mice

The animal experiments in this study were performed as approved by the animal experimentation ethics committee of the Peter MacCallum Cancer Centre. PTPRCA-Ly5.1^+^ or PTPRCA-Ly5.1/ly5.2 mice were purchased from WEHI, (Melbourne, VIC, Australia). Lgl1^+/−^ [6], TEL-JAK2 and EµMyc mice were bred and maintained at the Peter MacCallum Cancer Centre.

### Generation of chimeric mice

Timed matings were established between heterozygote polarity deficient animals. The following procedure was conducted on the day of harvest. Embryos were harvested from these matings at embryonic day 14.5 and genotyped by PCR on single cell suspensions from foetal livers. 8–14 week female PTPRCA-Ly5.1+ recipients received two doses of 5.5 Gy administered greater than three hours apart. 1×10^6^ of foetal liver single cell suspensions were injected intravenous (I.V). To prevent infection, reconstituted mice were maintained on neomycin sulfate–supplemented drinking water for 6 weeks post irradiation. 6–10 weeks post reconstitution peripheral blood was isolated from mice and successful reconstitution determined by percentage of Ly5.2+ cells.

### Analysis of peripheral blood

Automated cell counts were performed on blood collected from the retro-orbital plexus collected in eppendorf tubes containing a small volume of 20 mM EDTA. Samples were diluted 1∶12 with PBS and run on an Advia 2120 analyser (Siemens). For donor reconstitution and lymphocyte population analysis, peripheral blood was lysed to remove red blood cells then stained with antibodies against Ly5.2, B220, CD19, CD4 and CD8 (all purchased from BD). Samples were analysed on an LSR II flow cytometer (BD).

### Bone Marrow Transplantation

Whole bone marrow was isolated from femur and tibia of donor mice and transplanted as described in[17]. Briefly, recipient PTPRCA Ly5.1+ve mice received two doses of 5.5 Gy irradiation. Un-fractionated donor bone marrow was suspended in a balanced salt solution and administered intravenously to recipient mice. For long-term reconstitution assays, recipients received 1×10^6^ un-fractionated bone marrow cells. Donor and recipient cells were identified on the basis of Ly5.1/5.2 expression. For secondary transplants, Ly5.1/2 heterozygote mice were used as recipients to identify primary foetal liver donor cells and primary recipients from secondary recipients.

### Retroviral Notch introduction

For generation of Notch driven leukemia, Lgl1^+/+^ and Lgl1^−/−^ single cell suspensions were prepared from whole foetal livers isolated from 14.5 embryos. Suspensions were cultured in IL-3, IL-6, and stem cell factor conditioned media with 20% FCS for 3 days. Phoenix-E cells were transfected by calcium phosphate with MigR1 plasmids containing either GFP only or GFP with NotchICNΔRamΔP as described in [18]. Phoenix E cells were a gift from Marc Pellegrini (Walter and Eliza Hall Institute, Victoria, Parkville, Australia) and used as originally described [19]. Supernatants containing recombinant retrovirus were removed and used to transduce foetal liver cells. Recipient mice were prepared as described earlier and received 1×10^6^ GFP^+ve^ foetal liver cells by intravenous injection into the tail vein. Cohorts of reconstituted mice were the result of 2 independent foetal liver isolations and independent transfections.

### Monitoring mice for development of leukemia

Mice reconstituted with either NotchICNΔRamΔP transduced or EµMyc/TEL-JAK2 foetal livers were monitored daily for signs of leukemia onset or other signs of ill health. Mice were killed when deemed moribund by an experienced animal technician blinded to the genotype. Typically, mice presented with several of the following features: hunched posture, laboured breathing, weight loss, enlarged lymph nodes and/or spleen, peripheral white blood cell cellularity of 13×10^9^/L or greater. For Notch driven leukemias, peripheral lymphoid organs were analysed by flow cytometry for Ly5.2, GFP, CD3, CD4 and CD8 expression. For EµMyc driven leukemia, peripheral lymphoid organs were assayed for Ly5.2 and B220. For TEL-JAK2 leukemia, peripheral lymphoid organs were analysed by flow cytometry for Ly5.2, CD3, CD4 and CD8 expression.

### Real-time RT-PCR

RNA was prepared and real time quantitative PCR performed as described previously[20]. Primers used for PCR were as follows: Lgl1 Forward: ‘TCCGCATCATGGCCATCGGCACCA’;Lgl1 Reverse: ‘AGGCGGCCCTGACCAGGGAG G’; Lgl2 forward: ‘CGCTGCGCATCCTGGCCATCGG’ Lgl2 reverse: ‘GACCAGCTGACACTGACCAGGCAGGAA’. Samples were run in triplicate and mRNA expression of Lgl1 and 2 normalized to GAPDH using Rotor Gene 6000 Software (version 1.7). The relative expression levels to positive controls were calculated as 2^(−ΔΔCt)^.

### Statistics

Statistical differences between the means of two data groups was determined by using two-tailed unpaired Student's *t* test, and *p* values <0.05 were considered significant. For survival analysis, Kaplan-Meier survival curves were analysed using Log-rank (Mantel-Cox) test. P<0.05 were considered significant.

## Results

### Lgl1−/− foetal liver stem cells generate mature white blood cell populations

**Figure 1:**
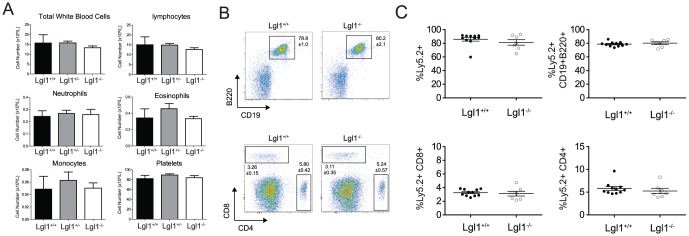
Analysis of peripheral blood in primary reconstituted Lg1^−/−^ mice. Lethally irradiated Ly5.1 mice were reconstituted with 1×10^6^ foetal liver cells isolated from E14.5 embryos from Lg1^+/+^, Lg1^+/−^ and Lgl1^−/−^ mice. (**A**) >6weeks post reconstitution, composition of the hematopoietic compartment was determined by ADVIA analysis of peripheral blood. (**B**) Donor derived Ly5.2^+^ lymphocyte populations in the blood of Lg1^+/+^ and Lgl1^−/−^ chimeric mice were identified by flow cytometry and frequencies quantified in (**C**). Statistics represent mean ± SEM from one reconstitution. Lgl*^+/+^ n = 4–11*, Lgl*^+/−^ n = 7–9* Lgl^−*/*−^ *n = 7–9*. One representative reconstitution is shown from 4 independent experiments

To investigate the role of Lgl1 in haematological malignancies, we generated chimeric mice by transplantation of E14.5 foetal liver cells from wildtype or Lgl1^−/−^ embryos into lethally irradiated recipients. Following transplantation, and as we previously observed [21], the composition of peripheral blood components and their numbers was not affected by loss of Lgl1 expression indicating that progenitors were capable of multi-lineage output (**Figure 1A**). As Lgl1 has been demonstrated to be a potent regulator of Notch signalling, we further characterized the composition of the white blood cell compartment and noted no difference in the frequency of lymphoid subsets between wildtype and Lgl1^−/−^ mice (**Figure 1B and C**). Interestingly, primary reconstituted mice displayed no signs of haematopoietic disease, even one-year post reconstitution.

### Transplantation of Lgl1−/− bone marrow does not induce leukaemia

**Figure 2:**
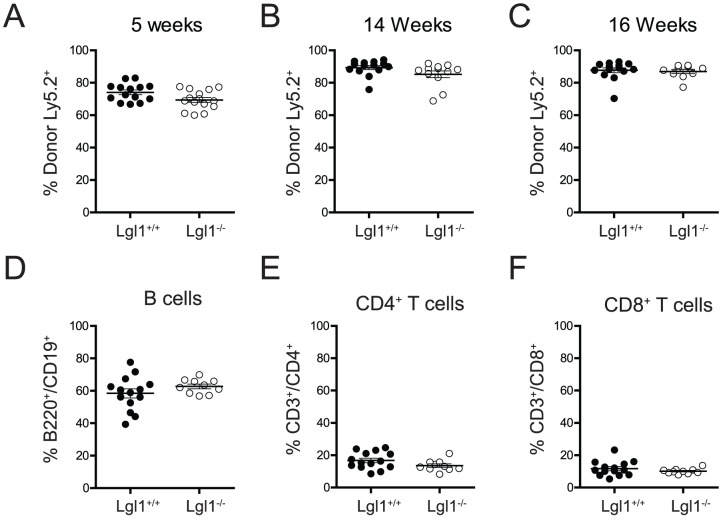
Long-term reconstitution with Lgl1^−/−^ bone marrow does not induce leukaemia. Unfractionated bone marrow was isolated from chimeric mice one-year post initial foetal liver reconstitution, and transplanted into Ly5.1/Ly5.2 heterozygote recipients. (**A–C**) Peripheral blood was analysed for the proportion of primary Ly5.2^+^ donor cells in peripheral blood at 5 weeks, 14 weeks, and 16 weeks post transplantation. (**D–F**) At 16 weeks, the lineage potential and existence of leukemic clones of transplanted bone marrow was assessed by measuring the proportion of primary donor Ly5.2+ B cells (B220^+^, CD19+), CD4 T cells (CD3^+^, CD4^+^) and CD8 T cells (CD3^+^, CD8^+^). Data shown are pooled results from 3 individual donors per genotype.

To examine whether Lgl deficiency increases the development of latent leukemic clones that would only become evident after extended periods of time, we transplanted bone marrow from mice one-year post foetal liver reconstitution into Ly5.1/2 heterozygote recipient mice and measured the production of notch dependent white blood cell lineages and their potential expansion. Reconstitution by Lgl1^−/−^ bone marrow cells was normal as measured by the proportion of donor cells in the peripheral blood at 5, 14 and 16 weeks post reconstitution (**Figure 2A–C**). Furthermore, at 16 weeks post reconstitution, peripheral Ly5.2^+^ T and B cell populations (that require strict regulation of Notch signalling) were present at normal frequencies (**Figure 2D–F**). These results were unexpected, and indicate that Lgl1 deficiency alone is not sufficient to induce leukaemia or perturb lymphoid development.

### Lgl1 does not modify disease progression in Myc, TEL-JAK and Notch driven leukaemia models

**Figure 3:**
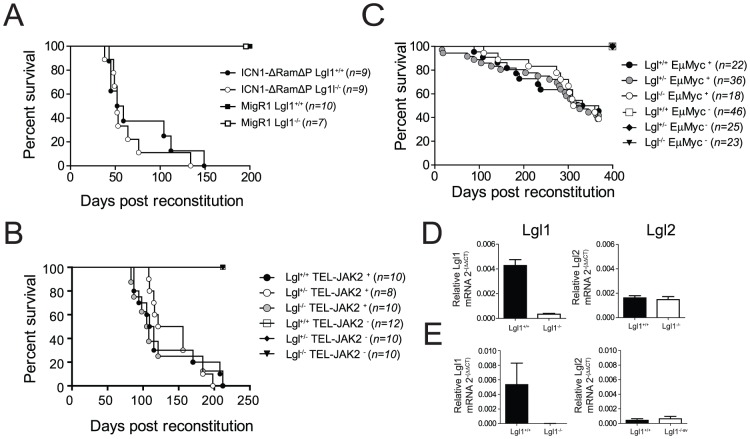
Lgl1 does not function as a tumour suppressor in conjunction with common oncogenes. Lg1^+/+^ and Lgl1^−/−^ foetal liver cells were infected with either empty vector MigR1-GFP or ICN1-ΔRamΔP-GFP constructs and transplanted into lethally irradiated Ly5.1 recipient mice. (**A**) Kaplan-Meier analysis of survival of mice following reconstitution with infected foetal liver cells. Kaplan-Meir analysis of survival in lethally irradiated Ly5.1 mice reconstituted Ly5.2+ Lgl1^+/+^,Lgl^+/−^ and Lgl^−/−^ foetal liver cells in the presence of either the TEL-JAK2 (**B**) or Eµ-Myc (**C**) transgenes. Splenocytes (**D**) or Eµ-Myc B cell lymphomas (**E**) were isolated from primary chimeric wildtype and Lgl deficient mice and assayed by Q-PCR for expression of both Lgl1 and Lgl2 in triplicate samples. Statistical differences between the means of two data groups was determined by using two-tailed unpaired Student's *t* test, and *p* values <0.05 were considered significant. For survival analysis, Kaplan-Meier survival curves were analyzed using Log-rank (Mantel-Cox) test. P<0.05 were considered significant.

We next investigated whether the tumour suppressor function of Lgl1 was capable of modifying disease progression in well-characterized mouse leukaemia models. As Lgl expression has been suggested to regulate Notch signalling [16], [22], we tested a model of T-ALL driven by constitutive Notch activation which can mimic the molecular status found in over 50% human T-ALL [14]. Additionally, we investigated how Lgl1 loss modified disease progression in leukaemia models driven by the commonly mutated oncogenes c-Myc and constitutively active JAK2 (in Eµ-Myc [23] and TEL-JAK2 [24] mice respectively). To test the function of Lgl1 in T-ALL, Lgl1^+/+^ and Lgl1^−/−^ E14.5 foetal livers were isolated from timed matings of Lgl1^+/−^ mice and infected with either a construct encoding intracellular notch modified to reduce oncogenic potential (ICN1-ΔRamΔP [18]) or an empty vector control (MigR1). For Eµ-Myc and TEL-JAK2, E14.5 foetal liver cells were generated from the respective transgenic lines by timed matings as described previously [17]. In all systems, 1×10^6^ foetal liver cells were transplanted into lethally irradiated Ly5.1^+^ recipients and survival and leukemia onset monitored. Remarkably, irrespective of the Lgl1 genotype of transplanted cells, we observed no significant change in latency in ICN1-ΔRamΔP – (Log rank Mantel-Cox test P = 0.5465), Eµ-Myc- (Log rank Mantel-Cox test P = 0.4607) or TEL-JAK2 induced leukaemia (Log rank Mantel-Cox test P = 0.9071 – **Figure 3A, B and C**). Furthermore, consistent with our previous results, and others [15], we did not observe development of leukaemia in Lgl^+/+^ or Lgl^−/−^ control chimeric mice. In adult mice, Lgl1 is expressed globally whereas the homologue Lgl2 is restricted to heart, kidney, liver, lung, skin, intestine and stomach. To eliminate the possibility that increased expression of Lgl2 compensated for loss of Lgl1 in the haematopoietic system, we isolated peripheral immune cells from primary chimeric animals and B cell lymphomas from Myc^+^ primary foetal liver chimeras and assayed for expression of Lgl1 and Lgl2 (**Figure 3D and E**). We observed no increase in the expression of Lgl2 suggesting that compensatory mechanisms were not responsible for maintenance of tumour suppression. Additionally, the basal levels of Lgl2 in wildtype mice were significantly reduced compared to Lgl1, consistent with characterisation of mouse expression patterns which illustrate Lgl2 is not expressed in peripheral lymphoid organs such as spleen or thymus [6], [25].

## Discussion

Lgl1 has recently been demonstrated to regulate HSC cell cycle and depletion of Lgl1 in HSC to confer a reconstitution advantage when in competition with wild type HSC [15]. Here, we demonstrate that in steady state haematopoiesis, downstream differentiation events from HSC reconstitution are intact. Namely, in the absence of Lgl1, production of all major blood lineages are unaffected. Therefore, the role of Lgl1 in the regulation of haematopoiesis described by Heidel et al might be restricted to HSC function and, not broadly applicable subsequent components of the haematopoietic system.

Interestingly, we observed no development of leukaemia in primary chimeric mice consistent with previous studies using conditional Lgl1 deletion [15]. Furthermore, non-competitive transplantation of bone marrow isolated from primary chimeric mice 1-year post reconstitution did not demonstrate any bias in lymphocyte populations (consistent with intact notch signalling in primary reconstituted mice during steady state haematopoiesis) and no expansion of latent leukemic clones from donor mice. In addition, we observed no modification of disease latency in the presence of common oncogenes. This included a modified version of intracellular notch (ICN1-ΔRamΔP) to reduce oncogenic potential, as well as co-operating mutations such as c-Myc and TELJAK2 which are sufficient to induce leukaemia on their own and susceptible to modification of disease in the presence of aberrant notch signalling. This is surprising, as gene signatures associated with loss of Lgl1 expression in mouse HSCs have been correlated with a poor prognosis in human AML [15]. However, the lack of tumour suppressor function observed here in B and T cell leukaemia is consistent with studies performed by delineating expression profiles of distinct human AML stem cell populations. These studies did not identify any significant expression differences of either Lgl1 or Lgl2 when compared to normal HSCs [26]. Recent studies have also demonstrated that deletion of aPKC-ζ, the essential upstream regulator of Lgl1 has a negligible effect on HSCs in both a steady state and competitive transplant situation [27]. Combined, these findings suggest that the role of Lgl1 in the haematopoetic lineage may be more complex than first thought. They suggest that the function of Lgl1 as a modifier of notch signalling and as a tumour suppressor might be restricted to a limited set of oncogenic lesions or alternatively, specific cellular contexts such as the HSC compartment.
